# The ILO and Transformation of Labour Law

**DOI:** 10.1007/978-3-030-55400-2_2

**Published:** 2020-10-17

**Authors:** Ulla Liukkunen

**Affiliations:** 1 Office of the President of the Republic of Finland, Helsinki, Finland; 2 Faculty of Law, University of Helsinki, Helsinki, Finland; Faculty of Law, University of Helsinki, Helsinki, Finland

## Abstract

The article explores some of the biggest challenges to the ILO caused by globalization and altering of the collective labour rights scene. It examines the recent transformation of collective bargaining regimes at national and transnational level and the consequences for normativities that characterize the relationship between labour law and the system of international labour standards. Domestic bargaining regimes are influenced by decentralization whereas in a transnational setting, with the phenomena of contractual arrangements between multinational enterprises and trade unions or other employee representatives, transnational collectivization of labour law is occurring. The process of transnationalization of labour law affects the traditional labour law paradigm with profound consequences for our understanding of the purpose and role of labour law. The transformation of labour law highlights regulatory developments that require reinforcement of the role of fundamental labour rights. Building a perspective on major global challenges to the ILO at the beginning of its second centenary requires an assessment of the labour question in terms of flexibility and vulnerabilities. This raises the question of inclusivity, calling for the ILO decent work agenda, employment creation, social protection, rights at work and social dialogue, all to be more firmly integrated in global regulatory approaches to work.

## Introduction

In a globalizing world, questions of working life remain central to most people.^1^ These questions place at the centre individuals and their well-being, and they offer perspectives of vulnerabilities to society that voices of the globalization of economy tend to sideline. But while norm-setting for international economic relations has been considered central to global governance, norm-setting for the governance of the social dimension of globalization has been greeted with less enthusiasm. Recently, we have been witnessing times where the economic transformation has led to accumulating challenges to labour protection that have turned out increasingly difficult to overcome. The objectives of the oldest UN agency, the ILO, have met obstacles that seem to flow directly from the way economic-political globalization has been writing the regulatory agenda for states.^2^

The effects on the labour market of globalization of the world economy have been altering the role of traditional regulatory actors. In recent decades, states and social partners, the actors that work tripartitely in the ILO towards social justice and international labour standards, have confronted changes in their position that have made them weaker builders of a better working life. States’ ability as regulatory actors has diminished as increasingly often they face situations beyond the reach of their legislative powers. For the social partners, declining coverage of collective agreements has meant changes in their position and regulatory power. Consequently, the role of labour law has also been changing. With a strong emphasis on economic competition, the post-industrial era has come to mark a profound change in conceptions of the objectives and functions to be set for labour law.

States have started to respond to global economic competition with regulatory strategies that tend to highlight flexibility over labour protection. This trend has gained support from international financial institutions that have long argued for flexibility and deregulation of the labour market.^3^ It has been suggested that labour law models need an adjusted framework to enable more competitive flexibility. Simultaneously there are demands for more inclusive regulatory approaches as old categorizations in labour law are building divides that strongly impair working individuals.^4^ The idea of employment contract-centred labour law has come under mounting question.^5^ Increasingly, not only the regulatory model of labour law but also its foundations, in terms of substance, sphere and institutions, have been put to the test with new labour market realities.

Against this background, the ILO as an organization appears to have been given an almost impossible task to promote social justice and fairer globalization.^6^ A weaker commitment to workers’ human rights seems to be a direct consequence of a global legal environment where the power of states as regulatory actors is not the same as hitherto. As the ILO’s governance model is based on tripartism, efforts on the part of the organization have become more challenging also with the development of the diminishing power of the social partners. We are witnessing both institutional and regulatory changes to the labour market that push forward decentralization of collective bargaining. The entire industrial relations infrastructure has been changing so that legal-institutional structures have been affected. Meanwhile, the transnational dimension of labour law has been evolving, providing a new normative basis for transnational industrial relations and cross-border collective contractual arrangements.

This article discusses some of the biggest challenges to the ILO caused by the altering scene of globalization and collective labour law regulation. It examines the recent transformation of collective bargaining regimes at national and transnational level and the consequences for normativities that characterize the relationship between labour law and the system of international labour standards. The transformation of labour law highlights developments that deserve attention when we consider the role of the ILO at the beginning of its second centenary.

## The Labour Question

Globalization and the changing nature of work and work organization have challenged national industrial relations systems and diminished the power of social partners. Ongoing development has strongly affected collective bargaining regimes and altered their nature. At the same time, the traditional regulatory approaches of labour law have been challenged even more broadly, as managing changes in working life—caused, *inter alia*, by globalization, migration, an ageing workforce, urbanization, platformization, digitalization, climate change, and, most recently, the covid-19 pandemic—poses a central dilemma to national systems that were originally built for a more stable labour market. Importantly, the interplay between industrial relations and collective bargaining is undergoing complex change.^7^

Managing changes in working life caused by globalization poses a central challenge to labour law.^8^ Protecting workers has become more difficult in a globalized world but there appears to be something even more fundamental in this dilemma: it is as if the understanding of those in need of protection offered by labour law would not be enough to produce socially just outcomes.^9^ The dilemma could be illustrated with some observations of narratives that demonstrate the legal landscape where national labour law systems navigate.

The idea of embracing flexibility as a regulatory pattern in labour law involves a narrative of economic demands-based regulatory approaches meeting the needs of companies but increasingly also of individuals who are willing and capable to exercise their autonomy in building their jobs and careers. States have increasingly begun to make more room for individual autonomy when developing regulatory strategies that aim at more flexible labour standards and bargaining regimes that favour local level solutions. These developments involve regulatory solutions that do not necessarily undermine the worker-protective dimension of labour law but are coupled with it in the search for better employability and labour resilience. However, in several labour law systems, striving towards greater flexibility has come to signal a strong individualization trend in regulation with adverse consequences for labour protection.^10^

Competition between states has advocated labour law reforms that pursue, to a variable extent, less binding regulation and more investment-friendly regulatory regimes. Importantly, economic considerations tend to be highlighted when seeking responses to globalization through adjustments to collective bargaining regimes. As a result, regulatory strategies that collective bargaining has traditionally offered in terms of developing social cohesion and equal labour standards are given more limited room.^11^

In its core, the labour question has been traditionally connected to unequal bargaining power and the need to level the imbalance between employer and employee.^12^ However, this point of departure is becoming too narrow and is increasingly seen as demonstrating the rigidity of the limits of labour law. From the perspective of vulnerabilities, another perspective can be presented on the labour question. This tells a narrative where individuals do not determine their own path but are trapped in adverse conditions.^13^ While flexibility is often pictured as being associated with freedom, capability, individual choices and future prospects, vulnerability looms in the context of the past and burdening, or exploitation, something to be managed or eliminated. Migrant and posted workers are vulnerable groups throughout the global labour market but there is also structural vulnerability that has to do with choices of exclusion and inclusion in the decisive regulatory frameworks.^14^

Globally, working life is characterized by a widening divide between those being protected and those not. The need to pay heed to those who require protection beyond the constellation of an employment contract means a need to identify development trends that do not become visible from the regulatory *façade* of societies. Two billion workers are working in the informal economy.^15^ Although there is diversity in the circumstances of these workers, many lack decent working conditions. The narrative of vulnerabilities is pointing to a growing gap in the socio-economic position of individuals. Globalization has furthered problems relating to transnational social dumping, and the labour dimension of human trafficking still remains largely unidentified.^16^ With forms of exploitation that do not have national borders, the narrative of vulnerabilities makes more inclusive regulatory responses highly significant to the ILO as an organization whose purpose is to promote social justice universally.^17^

## Labour Law and Its Embedded Normativities

For the standard-setting work conducted by the ILO, it is not a matter of indifference how we define labour law issues and on what foundations the protective sphere of labour standards is being set in state systems of labour law.^18^ In this sense, Western conceptions of labour law have not been fully at ease with the ILO in its global endeavours. First of all, labour law is traditionally a very domestically oriented field of law, featuring domestically oriented actors, policy objectives and interests. There is a broad consensus on the diversity of regulatory models employed in different countries. This has emphasized the contextuality of national labour law models in different economic and social settings.

 Secondly, there are differences between labour law systems that easily become visible in comparative enquiries and mark different orientations to some basic questions of labour law. The difference between collective autonomy and contractual autonomy marks a noteworthy division of labour law approaches in domestic settings. While the first highlights collective bargaining, the second places an employment contract in the central position.^19^ Collective bargaining is a cornerstone of several European labour law models, and the UK model shows itself as resting on contractual autonomy, with the legal role of collective agreements remaining voluntary.^20^

Along with well-established individual and collective dimensions, labour law has what could be characterized as a normative-institutional dimension illustrating labour market mechanisms that rest on particular institutional settings.^21^ Their mutual connectivity and their influence on norm creation and enforcement is—in many countries—highly significant. As a public law-related enterprise, labour law highlights institutional conditions for developing workers’ protection. It further emphasizes maintaining social peace and stability as important goals for social dialogue. Each dimension of labour law carries a systematic value but they can also be claimed to demonstrate embedded normativities which manifest themselves in legal practices highlighting the importance of viewing together substantive and procedural aspects of protection of workers.^22^ The latter aspect relates to legal regimes as enabling workers to choose to use their collective rights or not.^23^

For the ILO, all this poses a demand to adopt a carefully composed picture of labour law which is necessary to approaching and explaining labour market phenomena without setting aside contextual nuances and underpinning values. The capability to speak the language of labour law has influenced not only the success of regulatory strategy but also the gradually developed working methods of the organization. To establish international labour standards, national diversity has required a particular sensitivity from the ILO as a regulator. An unspoken prerequisite has been to build the standard-setting work from the beginning on a strong tripartite basis and also to involve labour market parties in monitoring work.

The picture of labour law would remain incomplete if an approach based on labour rights were not to be noted. It is often emphasized that freedom of association requires particular attention within this frame.^24^ For the ILO, the labour rights frame has provided an essential point of departure. The norm-setting structure of the ILO, together with its system of international labour standards, has heightened the special weight of social dialogue and collective bargaining, which forms an integral part of freedom of association, as highlighted by the ILO Constitution.^25^

The Philadelphia Declaration, as a part of the ILO Constitution, sets out the obligation to further effective recognition of the right of collective bargaining.^26^ In the field of collective bargaining, too, the ILO approach has been characterized by noting the diversity of national models that can build collective voice and capacity. Yet, at the same time, the organization has been clear with the key components of the right to bargain and the requirements to be set to the autonomous framework of bargaining. Although standard-setting work of the ILO involves issues that raise different opinions among the ILO member states and the social partners, the strength of the ILO’s approach has been connected to tripartite co-operation, which has made states and labour market organizations work together for certain goals. 

In essence, the vitality of the system of international labour standards is dependent on the way the core area of labour rights is defined and spelled out by the ILO. Although the ILO is a central builder of minimum protection for workers, the deepest layer deriving from the Constitution of the organization binds together rights at work and social development. In this pursuit, international labour standards that protect freedom, equality and safety of workers are associated strongly with legal action against any injustice at work. Lately, this approach has gained a reinforced global perspective.

## The ILO Vision of Decent Work: An Inclusive View of Work

The concepts of employee and contract of employment are used in different ways by domestic labour law systems in defining the scope of application of labour standards and highlighting the special nature of the relationship between employer and employee. However, as globalization has led to an increase in forms of work that are not covered by traditional labour law, the fact that work is increasingly carried out in diverse ways that fall between the spheres of work in an employment relationship and work as an independent entrepreneur has made connecting the idea of protection with a certain pre-determined legal status inadequate. Moreover, globally, forms of work that stand outside the official systems of societies constitute a largening group beyond any formal groupings.^27^ Informal work is a significant form of employment, particularly in developing countries.^28^

As labour law with its protective elements has been unable to keep up with the changes in ways of working, a large number of workers have become unprotected. Workers in different positions share the same vulnerabilities.^29^ As the ILO Constitution states that labour conditions must be improved, this requirement is not dependent on the form of work, be it work in an employment relationship or some other way of working.

Decent work, originally introduced by ILO Director-General Juan Somavia at the International Labour Conference in 1999, covers non-employment contract-based forms of work and work in the unofficial sector.^30^ When Somavia introduced the concept of decent work in the ILO it was constructed from four strategic objectives: promotion of rights at work; employment; social protection; and social dialogue.^31^ From the beginning, there was an emphasis on the mutual interconnectivity between these objectives.

As far back as 2000 the ILO began a programme on decent work to pioneer ways in which decent work can be effectively promoted and applied in ILO member countries.^32^ Two years later, a pilot programme was initiated for integrating decent work into the poverty reduction framework.^33^ These moves were followed by an expansion of measures which manifested the centrality of the decent work agenda to the ILO as a means of renewal and modernization.

The decent work concept emphasizes that the social rights of labour are universal.^34^ While highlighting this, the ILO can be read to affirm that the mechanisms of traditional labour law are alone insufficient to tackle the labour question in an inclusive way. Importantly, equality efforts behind the concept of decent work are based on the idea that employment cannot be separated from the quality of work.^35^

The ILO World Commission on the Social Dimension of Globalization adopted the idea of decent work as the basis of its proposals, and initiated decent work as the global goal of the multilateral system.^36^ The report of the World Commission, issued in 2004, is written in the spirit of the Philadelphia Declaration.^37^ The report reiterated the same concerns that were raised when the ILO was being founded and that can be found in its Constitution—poverty and inadequate labour conditions—and affirmed the importance of increasing the ILO’s authority as a way of managing globalization.^38^

According to the World Commission, the management of globalization requires procedures that promote relating economic growth more closely with social progress and sustainable development.^39^ The Commission paid critical attention to the imbalance in the world economy resulting from a fundamental imbalance between the economy, society, and polity. To correct this imbalance, the Commission stated that better institutional frameworks and policies are required. In particular, the imbalance between the economy and society has a detrimental effect on social justice. Global rules are not balanced because economic rules and institutions are stronger than social rules and institutions.^40^

The most important task of the World Commission was to suggest concrete measures for managing the social dimension of globalization. The central vision of the Commission was a globalization process that would put people first, respect human dignity and consider everyone equal.^41^ The Commission report highlights that the basic principles which must guide globalization are democracy, social equity, respect for human rights and the rule of law. Importantly, the Commission’s labour-related proposals emphasize the objective of decent work for all as a global point of departure. They also highlight core labour standards as the minimum set of rules for which respect should be strengthened in all countries.^42^

The World Commission pointed out four factors that together form the concept of decent work. These are full employment, social protection, fundamental rights at work and social dialogue. According to the Commission, the concept of decent work is based on the idea that the development of social and labour policies requires a balance between employee protection, job creation, and competitiveness.^43^

Before conceptualization of decent work, which has become central to ILO globalization policy, the strategy of the ILO rested on a different scheme which highlighted perspectives deriving from Western-embedded labour law settings that focus on employment contracts and their regulatory frame. With decent work, the ILO adopted an inclusive view of work, a view stemming from its Constitution.^44^ In so doing, the organization stressed the need to develop social and economic systems that guarantee basic security and employment but that also adjust to rapidly changing circumstances in a global market. The decent work agenda of the ILO builds on four pillars: (1) employment promotion, (2) social protection, (3) social dialogue and (4) rights at work. A synthetic perspective on the pillars has been strongly advocated by the ILO.^45^

The Declaration on Social Justice for Fair Globalization of 2008 was the outcome of tripartite consultations that began in the wake of the Report of the World Commission on the Social Dimension of Globalization. The Declaration institutionalized the decent work agenda, placing it at the core of the ILO’s efforts to reach its constitutional objectives. Freedom of association and effective recognition of the right to collective bargaining were held as particularly important to enable realization of decent work.^46^

In the UN 2030 Agenda for Sustainable Development, decent work is one of the indivisible sustainable development goals (SDGs), formulated as “Decent work and economic growth”. The wording of SDG 8 does not correspond to the ILO original point of departure and the goal of decent work was included in the Millenium Development Targets only in 2005. For the ILO, SDG 8 is, however, an achievement based on its efforts to have decent work adopted in the 2030 Agenda.^47^ Importantly, the idea of inclusivity was placed at the heart of sustainability. The UN targets for SDG 8 do not completely correspond to the decent work agenda of the ILO, and the perspective on fundamental labour rights advocated by the ILO would require broader attention.^48^ However, the UN 2030 Agenda has turned out in many ways important to the ILO, offering a longed-for opportunity to strengthen the position of the organization within the UN system.

With decent work, the ILO has gained a new voice within the UN system and the international multilateral system more generally. Thus, at the beginning of its second centenary the organization has acquired an opportunity to build more authority on its renewed character of global orientation. While the decent work agenda has gradually grown to renew the way of approaching the labour question in the regulatory strategy of the ILO, it can be seen as having potential to reinforce the global role of the organization. In essence, decent work as an objective rejects the narrow conceptual frame of traditional labour law, reminding us that labour law cannot be far from any groups of working individuals.

The ILO Centenary Declaration for the Future of Work of 2019 constructs a commitment to decent work and sustainability by linking social, trade, financial, economic and environmental policies together. It states that the ILO must move forward into its second century by further developing its human-centred approach to the future of work, which puts workers’ rights and the rights of all people at the heart of economic, social and environmental policies. Moreover, it highlights the decent work agenda.^49^ However, there seems to be a need for a perspective of broader interconnections between decent work and climate change within the sustainable development framework under construction. Although the ILO Guidelines for a just transition towards environmentally sustainable economies and societies for all,^50^ issued in 2015, present several ways of reinforcing these interconnections and “just transition” has been adopted as an objective of the ILO policy efforts towards sustainability, there would be a need for further elaboration.^51^ It is to be noted that the environmental perspective on SDG 8 is also still under construction.^52^ There appears to be a need for a broader approach which puts weight on a decent life as a frame for decent work to assess and alter societal processes that hinder humane conditions of work.

## Fundamental Labour Rights: Tasks Ahead

Fundamental labour rights, or core labour standards, form a central pillar of the concept of decent work.^53^ They also integrate decent work in the core of the system of international labour standards. The decent work agenda highlights social dialogue and collective labour rights in dealing with inequalities.^54^ On this view, the demand for decency derives from the demand for democracy and participation, aligning with the ILO Constitution.

However, efforts surrounding the definition, goals, and content of the core standards were originally met with considerable international debate and controversy, which lasted until the end of the 1990s. Prior to the definition of core labour standards in the 1998 ILO Declaration on Fundamental Principles and Rights at Work,^55^ a lack of clarity existed as to standards that could be considered central labour standards and whose global implementation should be promoted.

A central starting point for the 1998 Declaration was the UN World Summit for Social Development in Copenhagen in 1995. The Summit was the first time the social dimension of globalization was discussed at the highest political level. It approved the Copenhagen Declaration on Social Development and Programme of Action where governments agreed to promote the fundamental rights of employees. These were based on the central ILO conventions and included forced and child labour bans, freedom of association, collective bargaining rights, the principle of equal treatment of men and women, and a ban on discrimination.^56^ It was the first time the content of core labour standards was defined on the basis of the central ILO conventions. The Summit followed the first WTO Ministerial Meeting held in Singapore in 1996, which also played an important role in the development. The Meeting approved the Singapore Ministerial Declaration containing a commitment to observe internationally recognized core labour standards. The Declaration also expressed the WTO’s approval of the ILO’s activities and accepted that preparing international core labour standards fell under the ILO’s authority.^57^ It brought clarity to the role of the ILO and included clear approval by the WTO of core labour standards.^58^

The Declaration of the WTO Meeting in Singapore formed a central source in preparing the ILO Declaration on Fundamental Principles and Rights at Work, indeed affecting its content. The ILO Declaration was approved at the 86th Session of the International Labour Conference in 1998 and the content of core labour standards was exactly the same as when they were defined for the first time at the Copenhagen Summit. Essentially, the core labour standards were a development which followed the 1996 WTO ministerial meeting in Singapore, where it was affirmed that the ILO is the competent body to set and deal with these standards. The four principles in the 1998 ILO Declaration, generally referred to as fundamental labour rights or core labour standards, are: (1) freedom of association and collective bargaining, (2) prohibition of forced labour, (3) elimination of child labour and (4) non-discrimination in employment. These core labour standards are included in a total of eight ILO conventions also referred to as the core or key conventions.^59^

The ILO Declaration transformed the way labour rights are viewed internationally although there has not been consensus on what this has meant in legal terms.^60^ Core labour standards are considered to be binding on ILO member states directly on the basis of the ILO Constitution and the principles of these standards are included in several international human rights conventions. The monitoring of core conventions that core labour standards derive from differs from that of other conventions in that reports must be produced on core conventions annually, while most of the other conventions are reported on once every five years. The Declaration on Fundamental Principles and Rights at Work introduced the Follow-up procedure that promotes implementation of core labour standards and involves regular reports on the implementation of ratified conventions as well as a complaints procedure based on the ILO Constitution. In addition, reports are requested every year from countries that have not ratified the core conventions. The Declaration aims to take into consideration difficulties for developing countries to adopt international core labour standards on account of their lower stage of economic development. It stresses that core labour standards must not be used for promoting protectionist financial goals and that the Declaration must not be appealed to for such purposes.

Great economic, social and political differences of countries have had an impact on attitudes towards core labour standards, which have also been criticized for being too narrow in content and because concentrating on them can mean disregard for other central labour standards.^61^ Often, occupational health and safety have been highlighted as issues that would require priority along with the core standards. Although the absence of health and safety from core labour standards poses a difficulty, the linkage between collective labour rights and health and safety should be noted.

Collective labour rights build on capacities and participatory mechanisms that are important for ensuring safety and health at work. In addition to social dialogue at the workplace level, employee participation rights, information and consultation, contribute to developing health and safety. Health and safety management can be supported by an efficient employee participation system. The central idea behind collective labour rights is that they carry in them a capacity building character which enables regulatory development towards fairer terms and secure conditions of work.^62^ The influence of collective labour rights thus also highlights their relation to other labour standards.^63^

ILO Conventions Nos. 87 (freedom of association and the right to organise) and 98 (collective bargaining) set together the fundamental frame for collective labour rights. The evolution of the number of ratifications of core conventions, however, shows that significant progress is lacking in their adoption and recent regulatory development has also posed problems to these two conventions. To illustrate, the workforce of countries that have not ratified Convention No. 87 amounts to over 1.55 billion workers, and Convention No. 98 over 1.49 billion workers. Generally, protection of the right of freedom of association and collective bargaining has declined in recent decades.^64^ Notably, this kind of development is also visible in countries that have ratified these two core conventions.^65^

Adoption of the decent work agenda and the related strategy shift of the ILO can be seen as a kind of response to weak recognition of core labour standards and the relatively low number of ratifications of the recent ILO conventions.^66^ Even when conventions have been ratified, their enforcement has often been viewed inadequate. The 1998 Declaration is premised on the idea that core labour standards are global and they have been formulated to the effect that they can be universally applied.

The development of ratifications of ILO core conventions raises a need to call for a more precise account of fundamental rights development, aligning it to the broader frame of labour market and societal changes in each country. One could also ask whether the perspective that is traditionally offered to an assessment of the influence of fundamental labour rights on national labour law regimes needs complementing. A broader perspective would bring together different actors as participants of legal practices: legislatures, labour inspectorates, courts and arbitration committees as well as various non-state actors that shape and foster space for the evolution of labour rights in different and differentiated legal and societal contexts of work.^67^ There are countries that despite non-ratification of core conventions, often with long-term country-specific technical assistance from the ILO, strive towards developing their laws to meet the core labour standards. On a broader view, the effects of international labour standards can be found at different layers of normativity, which highlights interaction, dialogue and mutual enforcement.^68^

Of the UN organizations, the ILO is unique in its tripartite nature. This adds special weight to the organization’s standard-setting work and monitoring mechanism.^69^ Throughout its history, the ILO has carried out its work in different kinds of situations where the co-operation model has been put to the test. A recent development posing a new kind of challenge to the ILO comes from outside the classic labour standard-setting paradigm. This concerns a need to take a stand towards new regulatory actors entering the international labour standard-setting arena. The ILO is present in a supportive role in the social development of individual countries, but its authority and presence would also be required in transnational regulatory settings where new labour standards are being created—often with remarkable speed and intensity. There is a need to more firmly anchor respect for fundamental labour rights to the area of cross-border privatization of labour law. The question of effective incorporation of core labour standards into transnational sets of labour standards created by various non-state actors has become increasingly central in terms of labour protection and the social dimension of globalization. While promoting ratification of ILO conventions follows well-established operational modes, promoting transnational incorporation of core labour standards takes the ILO into unknown regulatory terrain. Yet the voice and authority of the ILO is needed in regulatory contexts where international actors independently organize and create new sets of rules of labour governance to ensure that the labour rights-based perspective is not sidelined.

## Collective Bargaining and Changing Regulatory Frames

Freedom of association and right to collective bargaining are facing challenges that derive from both international and domestic regulatory settings.^70^ Developments in the EU point to the vulnerability of domestic collective bargaining regimes along with a certain undermining of the right to exercise collective rights. There are many approaches to labour law in the EU, and the expansion of substantive EU labour legislation has improved labour protection in many central issues. However, as strong economic integration has been held as vital to the competitiveness of the internal market and EU Member States, it has resulted in overriding the respect for fundamental labour rights and values in the context of cross-border employment when the exercise of EU fundamental economic freedoms is involved. In the *Viking* and *Laval* cases, the CJEU handed down controversial judgments that demonstrate a tension between the right to collective bargaining, or the right to industrial action, on the one hand, and EU fundamental economic freedoms, on the other.^71^ These judgments inaugurated a new era of fundamental labour rights in the EU. Although the EU Charter of Fundamental Rights protects the right to bargain collectively, together with the right to industrial action, in these CJEU judgments collective labour rights have been subjected to certain limitations in a cross-border setting when fundamental economic freedoms are at stake but without a clear constitutional basis for this. The practice of the ILO supervisory organs does not recognize the type of discretion that the CJEU has applied in its jurisprudence concerning the conditions set to the right to take industrial action.^72^

 CJEU jurisprudence in the *Laval Quartet* has been challenging in terms of the workers’ human rights commitments of the EU Member States.^73^ Significantly, from the 1990s onwards, the ECtHR has developed the protective nature of the European Human Rights Convention and increasingly extended protection of the principles of the Convention to govern collective labour rights. To some extent, this has offered a counterforce to the destabilization of the protective framework of cross-border collective labour rights caused by the jurisprudence of the CJEU. In *Demir and Baykara*, the ECtHR confirmed that Article 11 of the ECHR governs the right to collective bargaining.^74^

Moreover, recent development of domestic collective bargaining regimes is highly significant in terms of understanding the pressing challenges of labour law. Strong centralized collective bargaining systems are traditionally considered as a Continental and Nordic European phenomenon. There are also well-established systems based on decentralized bargaining, like those of North America and Japan. Importantly, each collective bargaining model should be seen in the context of the country’s labour rights status.^75^ Although the right to collective bargaining enjoys constitutional recognition in several national legal systems this does not necessarily translate into heightened protection.

The challenge of combining flexibility and safety penetrates collective bargaining systems, and related balancing efforts have increasingly often unravelled to the benefit of flexibility. A shift towards more local level bargaining has been simultaneously occurring in many bargaining systems. Yet decentralization has occurred within remarkably diverse regulatory frameworks with different emphases and divergent locally-embedded solutions.^76^ In some countries, centralized models of collective bargaining have increasingly been replaced by decentralized ones, whereas in others the national or sectoral level still plays a key coordinative role.

Increasing pressure towards greater flexibility and tensions between flexibility and security appear common to bargaining systems worldwide. Mandatory minimum protection of workers has been weakened in both systems that are decentralized by nature and systems that have become increasingly decentralized.^77^ Several development trends are reshaping the subject matter of collective bargaining and narrow the protective sphere of collective agreements. Also the role of minimum protection afforded by the system of general applicability of collective agreements (*erga omnes*) in some states has been affected.

Several countries have altered their regulatory approach to collective bargaining in a situation where union density is declining and the coverage of collective agreements is diminishing. The hierarchy between collective agreements at different levels has changed and decentralization of bargaining structures has become a significant regulatory objective for many national legislatures. Opportunities to deviate from labour legislation and from upper level collective agreements have been enabled to a larger extent by local agreements. This has brought about new kind of local labour governance models.

There are national legislatures that have actively sought to promote flexibilization and decentralization of the collective agreement system through reforms that touch upon the core area of collective autonomy. In some cases the reforms have resulted in tension between sectoral or branch level and local level agreements, especially when strengthening the status of local level agreements at the cost of higher level agreements and their coordinative function has been sought. In addition, *in peius* deviations from mandatory labour legislation or higher level collective agreements by local agreements have been enabled or expanded in some countries.^78^ To a notable extent this has occurred against the legal tradition and basic labour law principles of these countries.^79^

Even when local agreements are concluded on the basis of the competence conferred by a sectoral collective agreement, local agreements may lead to highly differentiated rules between companies. Differentiation, which may continue within individual companies, treats issues that are traditionally regulated by collective agreements very differently. In some issues benefits can be reaped while in others a broader regulatory frame could be necessary.^80^ In some countries, decentralization has been partial and gradual and based on well-established tripartite law drafting processes whereas in others less balanced processes and outcomes have undermined the role of social dialogue. On the other hand, decentralization can be seen not only as a result of legislative reforms, but also of decreasing trade union density, changes in the power balance of bargaining and overall weakening of the role of the social partners. But in some cases, as the post-socialist countries in Eastern Europe demonstrate, it can also be given particular historical explanations.^81^

### Individualization in Decentralization of Collective Bargaining

The individualization trend in labour law is often offered with an explanation which relates to economically indispensable efforts to meet the needs of companies and individual workers, albeit from different angles. For companies, flexibility in different forms has become essential to ensure continuity of business and change management, and the increase of regulative flexibility is rooted in this demand. Workers’ perspective involves a broad range of issues including the influence of the spread of atypical employment. While a more individualistic regulatory approach relates to growing flexibility, there is a growing number of new categories of workers whose position differs from that of a traditional employee. Different and differentiated groups of non-standard workers tend to have less bargaining power, but they may also have less opportunities of attending to collective efforts to improve labour standards. Although evidence is available that in some countries social dialogue involves developing new strategies to improve protection of atypical work, the transformation of work is so profound that it adds pressure to adopt more inclusive bargaining frameworks.^82^

The development of bargaining regimes in the direction of more flexibility is creating new kinds of vulnerabilities. Decentralization leads to situations where issues that previously were negotiated between collective actors are increasingly decided between employer and employee at the workplace level. Individualized bargaining agendas reflect a growing emphasis on the employer–employee relationship in decollectivization of labour law. Local bargaining is increasingly enabling differentiation of the terms of employment on the basis of the needs of individual companies. As a result, locally bargained rules are more individualized than those in higher level agreements.^83^

New patterns and methods of setting terms of employment are evolving at the local level in a way that highlights both local procedures and bargaining as an individualized process between employer and employee. As a result, the procedural protection offered by traditional means of collective bargaining systems is declining. Existing dispute resolution mechanisms are required to show adaptability in dealing with labour standards deriving from new kinds of contractual arrangements. While local bargaining allows much discretion, employees need procedural safeguards in order to ensure a sufficient balance of workplace-level negotiations.

It appears that the traditional mode of collective bargaining has lost sight of some aspects of the labour market change.^84^ This change calls for developing institutional settings and local bargaining capacities to enable negotiations based on a more equal footing between the parties and it also calls for rethinking the substance. A clearer picture is needed of how local bargaining and employee participation could be integrated in order to advance the capacities of local bargaining. Connections which often exist between collective bargaining, on the one hand, and employee information and consultation, on the other, also speak for improving coverage of employee participation systems in terms of different forms of non-standard work.^85^

### Decollectivization of Industrial Relations

In many bargaining systems, both decentralized and centralized, declining collective agreement coverage and union density as well as institutional and regulatory changes are driving towards decollectivization of industrial relations. Even where decentralization has been organized, notable changes have occurred in the institutional settings of bargaining frameworks. In some systems, the position of trade unions in local level bargaining has been weakened so that they can be bypassed when local agreements are negotiated.

Importantly, the interplay between industrial relations and collective bargaining is in the process of change. Well-established labour institutions have often had a multi-level impact on the development of labour standards not only within labour law regimes but also in making more room for a labour rights frame in societies. Often, long-term promotion of the interests of workers and bargaining have been required to enable the birth of the collective agreements, which have provided legally enforceable minimum standards. Today, decentralization decreases the bargaining power of trade unions which has built collective capacities in an evolutionary way. 

Collective bargaining is increasingly understood as producing frameworks for individualized flexibility along with adjustments to labour standards required for ensuring employability, competitiveness and efficiency. At the local level, new patterns and methods of setting labour standards are evolving in a way which highlights employer discretion. These developments have occurred simultaneously with a certain polarization of labour markets. However, it should also be emphasized that the transformation of industrial relations which relates to decollectivization has occurred in various degrees and modes in different bargaining systems.^86^

### The ILO and the Challenge of Decentralization

National regulatory frameworks which were originally built to enable and maintain autonomous collective bargaining within the framework of corporatist arrangements have been increasingly transformed into frameworks which not only coordinate and manage but also set limitations on collective bargaining. This change derives from economic considerations that align businesses interests and state regulatory approaches or, in the case of the EU, international institutions exercising financial power, as the experience of the European semester demonstrates. As a result, less inclusive and less protective collective bargaining regimes are emerging, highlighting the adaptability of labour and the adjustability of the system. To illustrate, austerity measures adopted within the economic governance model of the EU have influenced the regulatory framework of collective bargaining and labour standards, in particular in Mediterranean countries.^87^ These measures have confronted a critical stand by the ILO Committee of Freedom of Association. In the case of Greece, the Committee noted significant interventions in the voluntary nature of collective bargaining and in the principle of the inviolability of freely concluded collective agreements.^88^

Some of the changes in collective bargaining regimes that we are witnessing derive from regulatory adaptation to profound changes in work while some come from a certain economization of labour law regimes. The scope and extent of protection that collective channels and institutions provide to workers are being challenged in ways that bear consequences for the protection of the rights of workers. In many countries, including those with well-established centralized or decentralized bargaining regimes, collective labour law mechanisms are in complex transition. The development of individualization and decollectivization appears distant from the original idea of collective bargaining related to workers’ collective pursuit of labour rights. It can be argued that the pursuit of greater flexibility has come to undermine the labour rights perspective and values that are manifested in workplaces in the right to bargain collectively.

The principle of labour protection upon which labour law is built presupposes collective actors and institutions that can exercise collective power and pressure in order to manifest and defend the collective interest of workers. In essence, a set of key values, democracy, interest representation and autonomy, is involved.^89^ However, it should be added that singling out and focusing on the collective interest is not alone enough to identify the labour question of our day. The regulatory framework for collective bargaining needs to be viewed from a broader perspective in search of a response to changes in the labour market. In the future, we may face new types of labour institutions or reformed institutions and regulatory frameworks that replace or complement those based on a more stable working life. Reforms are required to build legal-institutional space for the development of meaningful employee participation in our time and to achieve an adaptable system of labour governance. Reforms could also be called for in order to advocate regulatory models to tackle most pressing issues of inequality in novel ways. Collective bargaining regimes have tended to focus minor attention on some areas of labour law. They could assume a greater role in promoting gender equality and women’s position in and contribution to the labour market, workers’ employability and protection regardless of age, race or other categorizations as well as other issues where more effective safeguards would be necessary.^90^

When asking what role the ILO should assume in this particular transition context it has to be recalled that the organization is known for careful observance of working life development. It appears clear that the ILO is needed not only to speak for and explain the foundations of collective labour rights but also to increase our understanding of a normative development driven by changes in different regulatory surroundings with the broader labour rights scene in mind. The emphasis of social dialogue and collective labour rights has been central to understanding the well-being of workers as offered by the organization.

A deeper meaning of the right to collective bargaining in its distinctive characteristic is that it introduces collective enabling capacity to labour relations and development of labour standards.^91^ The idea of labour protection as a collective phenomenon has been legitimizing the autonomy of collective bargaining and the social partners in their relation to the state. This, in turn, has shaped the strong status of collective agreements in many labour law systems. What has happened recently is that labour governance has given way to economic governance. Adjustments to bargaining frameworks placing great importance on economic factors have narrowed the space of labour rights-oriented argumentation and values.^92^ There is a need to reconnect the requirement of labour protection to economic performance and productivity in order to achieve a broader understanding of mutual connectivity.^93^ This requires a deeper dialogue underlining the role of the ILO.

## Transnational Dimension of Labour Protection

Some of the biggest challenges to labour law relate to the sway of its assumption of territoriality, which forces a broadening of horizons beyond domestically oriented considerations to transnational developments in labour law.^94^ Actors such as international financial institutions have entered the arena of international labour standards creation, shaking traditional assumptions of regulatory power and authority. As a consequence, labour law has increasingly come to operate as transnational law beyond the traditional national—international labour law dichotomy, resulting in regulatory developments that both supplement and compete with traditional legal frameworks. Within transnational private regimes, labour rights are addressed in the context of self-governance and contractual arrangements that are not guided by public regulation. Private actors that lack a connection to the traditional system of international labour standards assume capability for shaping modes of labour standards within transnational normative frameworks where they would otherwise be absent.^95^

Globalization has raised several challenges for international labour law and poses a constant test of the legal applicability of international labour standards when multinational enterprises (MNEs) are operating on a transnational basis in various countries and regions. The regulatory framework of MNEs is manifold, consisting of multiple overlapping regimes with a reach broader than the law of national states. Important international documents providing guidelines for MNEs were already drawn up in the 1970s. A pioneer in providing guidelines for enterprises was the OECD, whose Guidelines were drawn to provide recommendations for MNEs and included in the Declaration on International Investment and Multinational Enterprises.^96^ A year later, the ILO presented the Tripartite Declaration of Principles concerning Multinational Enterprises and Social Policy (MNE Declaration),^97^ and the UN launched negotiations for guidelines for MNEs.^98^ At this stage, business was still largely considered to be a bipolar operation involving home and host states.

The second phase of laying down guidelines for MNEs, which began in the mid-1990s, stemmed from expanding globalization and the networking of business operations. Importantly, it was this phase that introduced the 1998 ILO Declaration and resolved the question of the content of core labour standards. The OECD published a widely revised version of its Guidelines in 2000 to match the ILO Declaration, and renewed the Guidelines again in 2011.^99^ In the same year, the UN Guiding Principles on Business and Human Rights were adopted.^100^ Later, core labour standards were included in the MNE Declaration and the incorporation of specific decent work issues occurred in 2017.^101^ In recent years we have witnessed significant reformulations of MNEs’ global production as well as a remarkable expansion of international documents that promote labour protection in the operations of MNEs. Today, all the major public international guidelines that seek to steer MNEs’ behaviour recognize the status of ILO fundamental labour rights.^102^

However, the voluntary approach to labour rights suffers from weaknesses. Corporate codes of conduct rarely include full references to core labour standards despite multiple international efforts to reinforce them. Although they were included in the OECD Guidelines and in the ILO’s MNE Declaration, we remain far from core labour standards forming the core of enterprise labour policies. The division of duties between states and MNEs as regards the execution of workers’ rights has become one of the central questions in managing the social dimension of globalization.^103^ As privatization of labour standard-making proceeds, an increasing need exists to find ways to place fundamental labour rights protection more directly at the core of corporate social responsibility (CSR). There is a need to concretize other aspects of the decent work agenda in the operations of MNEs as well.

### Transnational Collectivization of Labour Law

Transnational labour law has broadened the spectrum of collective contractual arrangements that relate to promotion of labour protection. Previously the domestic nature of collective bargaining systems was emphasized, and questions concerning the cross-border dimension of collective agreements typically arose either when a domestic collective agreement was made to concern work to be carried out abroad, or when the applicability of a domestic collective agreement to workers temporarily working abroad was to be resolved.^104^

Transnational agreements, falling outside the traditional categorizations and conceptualizations of labour law, are brought about within transnational normative frames that cross countries and regions. These agreements strive from certain normative-institutional settings of company-level industrial relations. Both international and domestic labour organizations as well as European Works Councils (EWCs) have been negotiating these agreements from the labour side with MNEs. Especially the role of EWCs, established in the EU countries on the basis of the EWC Directive^105^ for transnational information and consultation of workers in large Community-scale undertakings and groups of undertakings, has become significant in paving the way for transnational contractual arrangements promoting labour rights.

Generally speaking, transnational company agreements (TCAs) encompass a variety of forms of agreement concluded between an MNE on the one side and international or national trade union federations or other parties representing employees on the other side. European TCAs, which normally apply to an MNE and its subsidiaries in the European countries where the multinational operates, typically reflect issues that are of concern in the European labour market, such as anticipating and managing social changes concerning restructuring.^106^

International framework agreements (IFAs), in turn, are a specific group of transnational agreements. They are concluded between an MNE and global union federations, and other parties such as an EWC or a global works council representing workers, with a global reach. Often, IFAs seek to ensure respect for ILO core labour standards in MNE operations in all the countries where the company operates. However, despite international efforts to advance broader applicability in the operations of MNEs, they frequently lack governance over company supply chains.

IFAs derive firstly from centralized negotiating processes, which are dependent on functioning social dialogue at the MNE level, and secondly from sufficiently balanced employee representation within MNEs. Concluding these agreements requires certain reorganization of the regulatory power of trade unions at transnational level. As they appear, IFAs conceptualize the transnational context of social dialogue, which significantly differs from contexts of national systems and balancing processes that lie behind collective bargaining in domestic settings.^107^ From the perspective of workers’ organizations, IFAs can be seen as strengthening not only industrial relations but also the global union federations themselves.^108^ They have an important role in shaping transnational industrial relations and social dialogue.^109^ Altogether, transnational agreements can be viewed as offering a foundation for collectivization of labour law in a transnational setting.

Transnational agreements are not concluded in a void but in interaction with diverse normative regimes. This makes it important to pay heed to larger institutional and regulatory structures and their enabling character.^110^ These agreements have grown out of a need to ensure compliance with certain labour standards and basic social values, but many other normative dimensions have remained underexplored. For example, the impact of the broader normative frameworks of corporate governance on transnational contractual commitments has remained a largely unexamined area.^111^

Another important issue is how diverse domestic regulatory frameworks of collective bargaining influence transnational negotiations. National collective bargaining systems may involve regulatory, structural or institutional constraints that restrict development of transnational contractual arrangements, as demonstrated by Brazilian experience of efforts to conclude a transnational agreement. In a case where an IFA was negotiated, Brazilian single trade union system, permitting only one trade union at each bargaining level, became a major obstacle to successful transnational negotiations until a contractual model to overcome this was developed.^112^ The institutional bargaining frame at the national level may also influence the development of transnational negotiations so that transformation of labour unions is required. Japanese experience shows that a decentralized bargaining model of enterprise-based unions may affect entering into transnational negotiations so that transformation is required from industrial relations institutions to build transnational negotiating capacities.^113^

Several aspects of the content of IFAs deserve attention. In addition to focusing on fundamental labour rights, IFAs tackle other issues that are relevant to equality and expansion of substantive content of labour protection. They involve issues such as social dialogue, health and safety at work, career and skills development, training, anti-corruption, protection of personal data and internet policy. The expansion of labour issues governed is a noteworthy development in terms of labour standards coverage.

Although IFAs are associated with promotion of fundamental labour rights, the role of collective labour rights may be limited or absent. However, there is also evidence of opposite development. The first Spanish agreement covering a retail supply chain was the agreement between Inditex and IndustriAll Global Union, originally concluded in 2007.^114^ The objective of the agreement, which was renewed in 2014, is to ensure respect for human rights within the labour and social environment by promoting decent work throughout the supply chain. What makes this agreement exceptional is that it emphasizes the relevance of the freedom of association and the right to bargain collectively in improving labour protection within the supply chain. According to the agreement, these rights provide workers in the supply chain with mechanisms to monitor and enforce their rights at work.^115^ Lately, the agreement was renewed so that a global trade union committee was set up for implementation of the agreement at global level. In addition, the agreement sets out an establishment of joint training policies and programmes that involve the workers at Inditex factories and suppliers in order to make progress on the promotion of social dialogue and workplace equality.^116^

It is well known that problems of lack of monitoring and enforcement are a central challenge for IFAs. Although some IFAs include implementation and enforcement mechanisms, sometimes these agreements are loosely formed as a complementary part of the CSR documentation of an MNE. However, connections between IFAs and CSR strategies of companies vary, and a company-specific IFA may support company CSR strategy by concretizing it in social issues and boosting its enforcement.^117^ IFAs may transform the CSR policies of multinationals into more concrete and binding commitments. Many IFAs provide a complaints procedure for workers if a violation of workers’ rights as stated in the agreement occurs.^118^ These agreements are often based on the idea that any disputes or breaches of labour rights governed are handled in the company in cooperation with workers’ representatives. However, there is evidence of problems involved with the efficiency of company-specific dispute-settlement mechanisms. This raises a concern about the extent to which such agreements can be regarded as advancing transnational accountability without further developing their implementation and enforcement. A particular problem often lies in implementing IFAs in relation to suppliers.^119^ Often these agreements merely include a commitment to inform or encourage suppliers to respect the agreement or parts of it without stating the consequences of failure to do so. Moreover, although trade unions would seem to prefer monitoring compliance with IFAs by employees and trade unions themselves, related structures and resources are largely lacking.^120^

Altogether, the impact of IFAs on labour rights and protection remains limited but the potential involved cannot be overlooked. With advancing globalization and complex modes of global production, it is important that transnational agreements can be drawn to cover companies’ entire field of operations. They produce transnational normativities that derive from denationalised social dialogue based on particular normative-institutional development in a transnational setting. However, their efficient implementation would need further action and structural solutions at the international level.^121^ The ILO could play a central role in these efforts.

### Expansion of the Transnational Construction of Labour Standards

With privatization of labour standards-creation, several developments point to the expansion of regulatory approaches that influence labour protection and labour standards on a transnational level with a limited account of labour rights. These developments emphasize heterogeneous labour standards creation by widening number of non-state actors that claim regulatory authority. Transnational labour standards have evolved regardless of the traditional system of international labour standards, but they should be viewed against this system to evaluate their role. On the other hand, privatization development entails particular governance structures, as the Accord on Fire and Building Safety in Bangladesh from 2013 demonstrates. This agreement, designed to make safe the working environment for the Bangladeshi Ready Made Garment Industry, was made between retailers and global brands and national as well as international trade unions.^122^ The Bangladesh Accord is legally binding and has a specific governance model which involves the ILO. The regulatory framework created brings together global and local strategies of labour governance and seeks to facilitate cross-border social dialogue in a novel way.

In the framing of labour governance at transnational level, foreign trade agreements (FTAs) have gained noteworthy significance and visibility in setting goals that integrate pursuit of labour protection to trade and investment. For example, the recent EU Free Trade Agreement with Vietnam includes a sustainability chapter which governs (i) recognition of the beneficial role of decent work; (ii) facilitation of trade and investment in environmental goods and services, which are relevant for climate change; (iii) development and participation in voluntary initiatives and regulatory measures to establish high-level labour and environmental protection; and (iv) promotion of corporate social responsibility. As this agreement shows, the decent work goal has gained a foothold in recent developments of FTAs but with obvious imprecision. Although labour standards have found their way into trade agreement clauses the differentiation of outcomes is remarkable and their ultimate goal often blurred.^123^ There is also a trend of refraining from adding promotion of collective labour rights to FTAs. Generally, a vocabulary of labour standards instead of labour rights is preferred.^124^

In the transnational dimension, new kind of regulators and ways of regulating complement but also compete with regulatory approaches and contents advocated by the ILO. Moreover, they build a perspective on labour standards that leaves traditional labour rights frames in the shade. Importantly, the transnational dimension of labour protection is not only complementary to domestic and international approaches but also has its own normative setting from which it stems and evolves, fulfilling lacunas by creation of transnational normativities within labour law beyond state frontiers.

However, fundamental labour rights integration with transnational normative development poses a challenge to the ILO. In a transnational setting, social justice cannot be achieved merely through material regulation as the institutional space of regulatory power gains additional significance. Hence, more attention needs to be placed on the procedural and institutional dimensions of regulatory efforts in a transnational regulatory environment.

## Conclusion

The process of transnationalization of labour law affects the traditional labour law paradigm with profound consequences for our understanding of the purpose and role of labour law, consequences that derive from the growing significance of transnational norm-setting in a cross-border frame. In recent decades, normative developments have occurred that detach spatial dimension of labour protection from the territorial allocation of protection as the sole starting point.^125^ Despite legal ambiguity and diverse experience in different states, transnational agreements add new regulatory frameworks and mechanisms to collective labour law. They involve a new kind of enhancement of regulatory instruments developing collective rule-making capacities and a normative-institutional dimension of labour law in a cross-border setting.

In the Western portrait, labour law reflects a certain tradition and culture, and a strong influence from labour market organizations. Against this background, an assessment of the changing legal landscape of labour protection requires a contextual point of departure. However, globalization challenges this constellation and narrows its premises. Countries with a lower level of development cannot be left behind. The objective of decent work requires inclusive responses to global challenges of labour regulation.^126^ This challenges old models of labour law thinking and initiates a search for collective participation mechanisms that best fulfil this requirement.

Demands of flexibility posed to labour law have challenged old conceptual underpinnings and shaped the understanding of objectives set to labour standards, shifting the perspective from a labour rights-based frame towards a standards-based one. However, there is a growing need to pay heed to increasing vulnerabilities in order to develop ways to make regulatory frames of labour law more inclusive. This requires a shift back to labour rights-oriented thinking. As uncertainties are growing in the labour market, states need to reactivate in ensuring a better balance between flexibility and security. Simultaneously, instead of maintaining the level of formal categorizations a deeper account is required so that all who work or would like to work are taken at sight. This scene alters the perspective as to questions of exclusion and inclusion; and it should alter labour law talk too.^127^

We have a broadening picture of the ways in which decentralization is changing domestic collective bargaining regimes—a picture that draws attention to the basic functions of collective labour law. Work for fairer globalization has met one of its hardest setbacks in the area of collective labour rights, calling for the ILO to offer a clearer vision of the road ahead. There is a need to construct ways to develop regulatory responses that highlight not only economic necessities but also equality and protection for workers so that the objectives of collective bargaining are considered in terms of employability and competitiveness as well as in terms of labour protection and inclusivity. We should also seek to recognize the impact of the regulatory changes we are witnessing at the level of embedded normativities of labour law as this would better bring into the spotlight changes in the normative-institutional dimension of labour law.

The picture of the challenges to collective labour rights is different when their role in a transnational setting is viewed. The evolution of transnational negotiations and contractual arrangements at the level of MNEs has been an important development adding a new transnational layer to industrial relations.^128^ Despite uncertainties, conclusion of transnational agreements demonstrates transnational social dialogue and institutional development which contribute to promoting compliance with fundamental labour rights and international labour standards more generally. Transnational negotiations are capable of producing rights-based regulatory frames in a cross-border setting. However, core labour standards should be more firmly included in these developments. In transnational regulatory developments, much work remains to pursue the commitment to core labour standards. As the status of fundamental labour rights is particularly weak in cross-border settings, the international commitment to foster regulatory development building on these rights would require a renewed role for the ILO. In order to deal with vulnerabilities that escape the state law frame, the ILO decent work agenda should include a transnational dimension.

While historical explanations of the evolution of labour law in industrial societies can be built with a focus on domestic labour law models, the world of work has been so strongly affected by globalization that an isolated evaluation of individual national-level regulatory frames for labour protection has become inadequate. The risk of inequality, unemployment and poverty is an essential threat to every society, and labour standards are meant to offer a buffer against reduced protection.^129^ In essence, any sketch of the labour question of our time has a global face.^130^

Importantly, the regulatory frame of sustainable development based on the key elements of the ILO decent work agenda, employment creation, social protection, rights at work, and social dialogue, should be more firmly integrated into global perspectives of the labour question if it is to work. Within this frame, a more concrete regulatory pursuit initiated by the ILO would send strong signals although it would require adopting the goal of a decent life as a frame to address situations which hinder decent work.

Still, even in fostering these pursuits, regulatory strategies that are based on decent work provide a solid point of departure only if fundamental labour rights are strengthened. Decent work connects the fight against inequality to the social dialogue and enabling the collective voice of workers. As such, it brings together the core content of the system of international labour standards and aspirations deriving from the origins of the ILO.

## Footnotes

1 As the ILO Commission of the Social Dimension of Globalization stated in its report, globalization affects people most directly through their work. See World Commission on the Social Dimension of Globalization (2004), p 64.

2 For a critical account of the imbalance from a labour law perspective, see Maupain (2013).

3 See, for example, Weiss (2013), p. 7.

4 Critically, see Langille (2019a).

5 See Davidov (2002).

6 See Maupain (2013).

7 See Liukkunen (2019a), p. 6.

8 See Liukkunen (2005).

9 See Langille (2018), pp. 101–103.

10 Liukkunen (2019a).

11 See Ibid., p. 40.

12 As characterized, “[t]he main object of labour law has always been, and we venture to say will always be, to be a countervailing force to counteract the inequality of bargaining power which is inherent and must be inherent in the employment relationship”. See Davies and Freedland (1983), p. 18.

13 The complexity of the dilemma of migration in relation to labour law has been observed, *inter alia*, by Costello and Freedland (2014).

14 On patterns of social exclusion and inclusion see, for example, Carr and Chen (2004). On migrant workers’ regulatory dilemma see Wolff (2018); and on posted workers’ regulatory dilemma see Chen and Liukkunen (2019).

15 See ILO (2018), p. 13.

16 See for example, ILO (2020) demonstrating this. See also ILO (2017).

17 See also Scelle (1930), p. 31.

18 See also Langille (2019a), p. 508.

19 See also Bogg et al. (2015), p. 4.

20 See also Collins (2015).

21 It should be noted that each division of labour law into different branches is shaped by legal-cultural characteristics. Even where the often used basic division of labour law into individual and collective is accepted, there is variation in the meaning content given.

22 Tuori makes a useful distinction between law and legal practices. See Tuori (2016), p. 6, where legal practices are defined as social practices specialized in the production and reproduction of law.

23 See also Langille (2018), p. 94, emphasizing the essence of the external legal structures labour law provides to bargaining regimes.

24 See Bogg (2015), p. 105.

25 See Constitution of the ILO which was adopted by the Peace Conference in April 1919, and became Part XIII of the Treaty of Peace of Versailles (28 June 1919). The Constitution has been amended in 1922, 1945, 1953, 1962, 1972, 1986 and 1997.

26 Several ILO conventions and recommendations of the ILO concern collective bargaining. See Convention concerning Freedom of Association and Protection of the Right to Organise (No. 87) adopted on 17 June 1948; Convention concerning the Application of the Principles of the Right to Organise and to Bargain Collectively (No. 98) adopted on 8 June 1949; Declaration on Fundamental Principles and Rights at Work. Adopted by the International Labour Conference, 86th Session, Geneva, 18 June 1998 (Annex revised 15 June 2010); Collective Agreements Recommendation (No. 91) adopted on 29 June 1951; Convention concerning Protection and Facilities to be Afforded to Workers’ Representatives in the Undertaking (No. 135) adopted on 23 June 1971; Voluntary Conciliation and Arbitration Recommendation (No. 92) adopted on 29 June 1951; Rural Workers’ Organisations Recommendation (No. 149) adopted on 23 June 1975; Convention concerning Protection of the Right to Organise and Procedures for Determining Conditions of Employment in the Public Service (No. 151) adopted 27 June 1978; Labour Relations (Public Service) Recommendation (No. 159) adopted on 27 June 1978; Convention concerning the promotion of collective bargaining (No. 154) adopted on 19 June 1981; Collective Bargaining Recommendation (No. 163) adopted on 19 June 1981.

27 See ILO (2001, 2002a).

28 See Daza (2005), where diversity of approaches to informal economy and its conceptualization as well as diverse treatment of informality are pointed out.

29 See Davidov (2002), p. 417.

30 See ILO (1999a).

31 Ibid.

32 The eight countries selected for the Programme were Bahrain, Bangladesh, Denmark, Ghana, Kazakhstan, Morocco, Panama and the Philippines. See ILO (n.d.-b).

33 Awad (2005), para 4.

34 See also Hepple (2002), pp. 255–256.

35 See ILO (1999a).

36 See World Commission on the Social Dimension of Globalization (2004), p. ix.

37 The spirit of the Philadelphia Declaration of 1944 is often recalled by labour law scholars. See also Supiot (2012).

38 See World Commission on the Social Dimension of Globalization (2004). See also ILO (2004).

39 See World Commission on the Social Dimension of Globalization (2004), p. 2.

40 See Ibid., pp. 3–4.

41 See Ibid., pp. 5–6.

42 See Ibid, p. 55, 91 and 110.

43 See Ibid., pp. 64–67 and pp. 108–114.

44 The Philadelphia Declaration of 1944, which forms an essential part of the ILO Constitution, extended the work of the ILO by stating that all human beings, irrespective of race, creed or sex, have the right to pursue both their material well-being and their spiritual development in conditions of freedom and dignity, of economic security and equal opportunity. It stated that the attainment of the conditions in which this shall be possible must constitute the central aim of national and international policy. See also Article 23 of the Universal Declaration of Human Right. Adopted by the United Nations General Assembly, 3rd Session, Paris, 10 December 1948.

45 See also ILO (1999b).

46 Preface to the ILO Declaration on Social Justice for a Fair Globalization. Adopted by the International Labour Conference, 97th Session, Geneva, 10 June 2008.

47 See Frey and MacNaughton (2016), pp. 2–3.

48 See UN (n.d.). On the other hand, although the decent work agenda governs only core labour standards explicitly, several ILO conventions relate to and support the agenda. See also MacNaughton and Frey (2011), p. 446.

49 See ILO, Centenary Declaration for the Future of Work. Adopted by the International Labour Conference, 108th Session, Geneva, 21 June 2019.

50 See ILO (2015).

51 See also Doorey (2017), Doorey (2015), pp. 560–563.

52 See for example the outcome of Weitz et al. (2019) pointing to difficulties of individual states in gaining an understanding of the environmental issues under SDG 8.

53 See Javillier (2003), p. 3.

54 See also Moreau (2013).

55 ILO, Declaration on Fundamental Principles and Rights at Work. Adopted by the International Labour Conference, 86th Session, Geneva, 18 June 1998 (Annex revised 15 June 2010).

56 See in more detail Copenhagen Declaration on Social Development and Programme of Action of the World Summit for Social Development 1995.

57 See WTO, Singapore Ministerial Declaration, adopted on 13 December 1996. See also Fields (2003), p. 65.

58 See Leary (1997), p. 1.

59 These conventions are the Convention concerning Freedom of Association and Protection of the Right to Organise (No. 87) adopted on 17 June 1948; the Convention concerning the Application of the Principles of the Right to Organise and to Bargain Collectively (No. 98) adopted on 8 June 1949; the Convention concerning Forced or Compulsory Labour (No. 29) adopted on 10 June 1930 (and its of 2014 to the Forced Labour Convention adopted on 28 May 2014); the Convention concerning the Abolition of Forced Labour (No. 105) adopted on 5 June 1957; the Convention concerning Minimum Age for Admission to Employment (No. 138) adopted on 6 June 1973; the Convention concerning the Prohibition and Immediate Action for the Elimination of the Worst Forms of Child Labour (No. 182) adopted on 1 June 1999; the Convention concerning Equal Remuneration for Men and Women Workers for Work of Equal Value (No. 100) adopted on 6 June 1951; the Convention concerning Discrimination in Respect of Employment and Occupation (No. 111) adopted 4 June 1958.

60 See for example Alston (2004), pp. 457–521 for the characteristics of the well-known scholarly criticism of core labour standards.

61 See for example Alston and Heenan (2004).

62 See ILO (2002b).

63 Ibid., p. vi.

64 See ILO (n.d.-a).

65 See Liukkunen (2019a). See also Marx et al. (2015).

66 As regards most recent conventions, the number of ratifications can be found at ILO (n.d.-a).

67 See an analysis of ways to approach the question of implementing international labour standards from a legal-cultural perspective, Liukkunen and Chen (2016), pp. 6–9.

68 See Liukkunen (2019a), p. 10.

69 See also in this volume Waas (2020).

70 Chapters 6 and 7 of this article draw partially on the author’s research published in Liukkunen (2019b).

71 CJEU (Grand Chamber), *Laval un Partneri Ltd v Svenska Byggnadsarbetareförbundet, Svenska Byggnadsarbetareförbundets avdelning 1, Byggettan and Svenska Elektrikerförbundet* (Case C-341/05), Judgment, 18 December 2007; CJEU (Grand Chamber), *International Transport Workers’ Federation and Finnish Seamen’s Union v Viking Line ABP and OÜ Viking Line Eesti* (Case C-438/05), Judgment, 11 December 2007.

72 See ILO Committee of Experts (2010), p. 209.

73 The Laval quartet constitutes of CJEU judgments Viking and Laval as well as CJEU (Second Chamber), *Dirk Rüffert v Land Niedersachsen* (Case C-346/06), Judgment, 3 April 2008; and CJEU (First Chamber) Commission of the European Communities v Grand Duchy of Luxemburg (Case C-319/06), Judgment, 19 June 2008.

74 See European Court of Human Rights (Grand Chamber), *Demir and Baykara v. Turkey* (App. No. 34503/97), Judgment, 12 November 2008.

75 See Liukkunen (2019a), pp. 4–5.

76 See Ibid., p. 5.

77 See Ibid.

78 See for example Kun (2019), Magnani (2019) and Mazuyer (2019).

79 See Liukkunen (2019a), pp. 16–20.

80 The ILO (2002b) points to the relevance of the level of bargaining in terms of health and safety issues. While much of the capacity building of health and safety protection occurs at the enterprise level, there are issues of health and safety where a national or sectoral level regulatory framework is needed to ensure necessary protection.

81 See Liukkunen (2019a), pp. 7–9.

82 Ibid., p. 54.

83 Ibid., p. 32.

84 See also Estlund (2015), p. 260.

85 Liukkunen (2019a), pp. 54–55.

86 See also Dukes (2014), p. 9.

87 See Liukkunen (2019a), pp. 29–31; Seifert (2014).

88 ILO Committee of Freedom of Association (2012), para 995.

89 See Liukkunen (2019a), p. 60.

90 See Ibid., p. 60.

91 See also Langille (2019b).

92 Liukkunen (2019a), p. 54.

93 See also in this volume Waas (2020).

94 See Mundlak (2009).

95 See Liukkunen (2014) pp. 163–167.

96 See OECD, Declaration on International Investment and Multinational Enterprises. First adopted in 1976, and reviewed in 1979, 1984, 1991, 2000 and 2011.

97 See ILO, Tripartite Declaration of Principles concerning Multinational Enterprises and Social Policy. Adopted by the Governing Body, 204th Session, Geneva, November 1977, and amended at 279th (November 2000), 295th (March 2006) and 329th (March 2017) Sessions.

98 The outcome of the negotiations was a Draft United Nations Code of Conduct on Transnational Corporations. For more details, see Intergovernmental Working Group on a Code of Conduct (1982).

99 See OECD (2011) OECD Guidelines for Multinational Enterprises. OECD Publishing, Paris.

100 Human Rights Council (2011).

101 See ILO, Tripartite Declaration of Principles concerning Multinational Enterprises and Social Policy. Adopted by the Governing Body, 204th Session, Geneva, November 1977, and amended at 279th (November 2000), 295th (March 2006) and 329th (March 2017) Sessions.

102 See Liukkunen (2016), p. 157.

103 See also ILO, OECD, IOM, UNICEF (2019), p. 1.

104 See Liukkunen (2019a), p. 44.

105 See Directive 2009/38/EC of the European Parliament and of the Council of 6 May 2009 on the establishment of a European Works Council or a procedure in Community-scale undertakings and Community-scale groups of undertakings for the purposes of informing and consulting employees (Recast).

106 See Liukkunen (2019a), pp. 44–45.

107 See also Liukkunen (2019a), p. 44.

108 See Müller et al. (2008).

109 See (2019a), pp. 44–49. See also Blasi and Bair (2019), p. 40; Papadakis et al. (2008), p. 85.

110 See Liukkunen (2013).

111 On the normative activities of multinationals and the legal environment thereto, see, Danielsen (2005), p. 412.

112 See Maia (2019), p. 118.

113 See Araki (2019), pp. 393–395.

114 See IndustriAll Global Union (2014).

115 See Chacartegui (2019), pp. 547–548.

116 See Global Framework Agreement between Industria de Diseño Textil, S.A. (Inditex, S.A.) and IndustriAll Global Union on the implementation of International Labour Standards throughout the Supply Chain of Inditex. Available at: http://www.industriall-union.org/sites/default/files/uploads/documents/GFAs/signed_gfa_inditex_-_english.pdf. Accessed 29 April 2020.

117 See Liukkunen (2014).

118 See Liukkunen (2019a), pp. 49–53.

119 See Blasi and Bair (2019).

120 See Liukkunen (2019a), p. 53.

121 See Liukkunen (2014).

122 See the Accord on Fire and Building Safety in Bangladesh, 2013. Available at: https://admin.bangladeshaccord.org/wp-content/uploads/2018/08/2013-Accord.pdf. Accessed 29 April 2020; and the Accord on Fire and Building Safety in Bangladesh, 2018. Available at: https://admin.bangladeshaccord.org/wp-content/uploads/2018/08/2018-Accord.pdf. Accessed 29 April 2020.

123 See also Banks (2011), pp. 48–49.

124 See Brown (2015, 2016).

125 See Mundlak (2009).

126 See also Hepple (2005), p. 19.

127 See for example Alan Bogg and Cynthia Estlund proposing novel ways to view and broaden the sphere of the right to the freedom of association, in Bogg and Estlund (2014).

128 Liukkunen (2019a), p. 55.

129 The Philadelphia Declaration of 1944 states that poverty anywhere constitutes a danger to prosperity everywhere and that the war against want must be fought as an international effort where representatives of workers and employers are equal participants along with government representatives.

130 The renewed universal approach of the ILO is manifested, for example, in the most recent Convention concerning the Elimination of Violence and Harassment in the World of Work (No. 190) adopted on 21 June 2019, which governs all workers regardless of the form of employment. See Article 2 of the Convention.
